# The Emotional Effect of Background Music on Selective Attention of Adults

**DOI:** 10.3389/fpsyg.2021.729037

**Published:** 2021-10-04

**Authors:** Éva Nadon, Barbara Tillmann, Arnaud Saj, Nathalie Gosselin

**Affiliations:** ^1^International Laboratory for Brain, Music and Sound Research (BRAMS), Music, Emotions, and Cognition Research Laboratory (MUSEC), Center for Research on Brain, Language and Music (CRBLM), Montreal, QC, Canada; ^2^Department of Psychology, University of Montreal, Montreal, QC, Canada; ^3^Center for Interdisciplinary Research in Rehabilitation of Metropolitain Montreal (CRIR), Montreal, QC, Canada; ^4^Lyon Neuroscience Research Center, CNRS, UMR 5292, INSERM, U1028, University Lyon 1, Lyon, France

**Keywords:** selective attention, inhibition, Stroop task, background music, musical emotion, background noise, arousal, neuropsychology

## Abstract

Daily activities can often be performed while listening to music, which could influence the ability to select relevant stimuli while ignoring distractors. Previous studies have established that the level of arousal of music (e.g., relaxing/stimulating) has the ability to modulate mood and affect the performance of cognitive tasks. The aim of this research was to explore the effect of relaxing and stimulating background music on selective attention. To this aim, 46 healthy adults performed a Stroop-type task in five different sound environments: relaxing music, stimulating music, relaxing music-matched noise, stimulating music-matched noise, and silence. Results showed that response times for incongruent and congruent trials as well as the Stroop interference effect were similar across conditions. Interestingly, results revealed a decreased error rate for congruent trials in the relaxing music condition as compared to the relaxing music-matched noise condition, and a similar tendency between relaxing music and stimulating music-matched noise. Taken together, the absence of difference between background music and silence conditions suggest that they have similar effects on adult’s selective attention capacities, while noise seems to have a detrimental impact, particularly when the task is easier cognitively. In conclusion, the type of sound stimulation in the environment seems to be a factor that can affect cognitive tasks performance.

## Introduction

Music is considered among the most enjoyable and satisfying human activities (Dubé and Le Bel, 2003). The recent development of portable players with unlimited access to musical libraries means that people’s access to music has never been greater than in the last decade (Krause et al., 2014). Adults listen to music for an average time of 17.8 h per week (International Federation of the Phonographic Industry, 2018). It is therefore possible to infer that most adults perform a large part of their daily tasks in the presence of background music (cooking, driving, working, studying, etc.). The efficient accomplishment of these tasks recruits the capacities of selective attention, also referred as attentional control; the cognitive ability to select, among a considerable load of information, relevant stimuli while inhibiting others (Murphy et al., 2016; Bater and Jordan, 2020). Due to their front-line role in information processing, selective attention capacities represent the gateway to other executive and memory functions, the latter allowing us to adapt to the demands of daily life (Cohen, 2011; Nobre et al., 2014). According to the preceding definitions, the presence of inattention would cause a deleterious effect on overall cognitive performance due to the processing of information irrelevant to the accomplishment of a task, at the expense of relevant information (Baldwin, 2012). Therefore, with a growing body of research showing that the presence of background music influences cognitive functioning (for review, see Kämpfe et al., 2010), it is important to better understand the influence of background music on selective attention. Particularly, this would allow for the development of recommendations aiming to optimize efficient performance in everyday life.

Research investigating the effects of background music on selective attention performance has shown mixed results; sometimes showing neutral (Petrucelli, 1987; Cassidy and MacDonald, 2007; Wallace, 2010; Speer, 2011; Cloutier et al., 2020; Deng and Wu, 2020), beneficial (Amezcua et al., 2005; Darrow et al., 2006; Cassidy and MacDonald, 2007; Masataka and Perlovsky, 2013; Slevc et al., 2013; Fernandez et al., 2019), or deleterious (Masataka and Perlovsky, 2013; Slevc et al., 2013; Fernandez et al., 2019; Deng and Wu, 2020; Cloutier et al., 2020) effects on performance. However, multiple factors can influence this variability, such as the methodological limits observed within this literature. Several studies present small samples of adult participants, making it difficult to generalize the results to the general adult population (≤24 adult participants; Amezcua et al., 2005; Speer, 2011; Giannouli, 2012; Fernandez et al., 2019; Cloutier et al., 2020). In addition, most of the time, non-auditory (e.g., silence) and auditory (e.g., noises with sound characteristics similar to those of music) control conditions were lacking (Darrow et al., 2006; Marchegiani and Fafoutis, 2019; Deng and Wu, 2020; Cloutier et al., 2020). There were also methodological limitations regarding the choice of the sound material used. For example, some studies have presented music with words (e.g., Darrow et al., 2006; Speer, 2011; Marchegiani and Fafoutis, 2019; Deng and Wu, 2020), which has generally resulted in a deleterious effect on performance. However, several studies have previously shown that the presence of speech or words in a sound environment tends to negatively affect cognitive performance in comparison with a speechless sound environment (e.g., Salamé and Baddeley, 1989; Szalma and Hancock, 2011). Therefore, the effect of language processing is confounded with the effect of background music in these studies.

Another element that could explain the variability between the results of previous studies is the lack of control over the emotional characteristics of the sound stimuli being utilized (as discussed in Kämpfe et al., 2010; Schellenberg, 2013). Indeed, different sound environments can induce different emotions. Particularly for musical stimuli, musical parameters, such as tempo, can be modulated to induce different musical emotions, like the level of arousal (Gabrielsson and Juslin, 2003); music with fast tempi are usually considered as stimulating, while music with slow tempi are considered as relaxing (Västjäll, 2001; Bigand et al., 2005; Vieillard et al., 2008). The emotional characteristics of a sound stimuli, like its level of arousal, are important to consider as studies have shown links between them and performance on cognitive tasks (e.g., spatial skills, Thompson et al., 2001; selective attention, Ghimire et al., 2019). Indeed, according to the arousal-mood hypothesis (Nantais and Schellenberg, 1999; Thompson et al., 2001, 2011; Husain et al., 2002; Schellenberg and Hallam, 2005; Schellenberg et al., 2008), cognitive performance can be promoted by sound stimulation, notably by increasing physiological activation and improving mood. Both music and noise can induce emotions (Hunter and Schellenberg, 2010), but there is a general agreement that music is efficient to induce positive emotions, and therefore it can be employed to positively modulate mood (Thompson et al., 2001). The previously cited research by Schellenberg and Weiss (2013) has shown that when participants listen to music that positively alters their mood before performing a cognitive task, like a stimulating and pleasant music, their performance in this cognitive task was improved. The arousal-mood hypothesis has been built on data that are based on listening to a stimulus before the accomplishment of a cognitive task.

The objective of this current study was to investigate the effect of the arousal level of background music on adults’ selective attention. To do so, we compared the effect of stimulating and relaxing music on performance at a Stroop-type task, with two music-matched noise conditions (stimulating music-matched-noise and relaxing music-matched noise), and a silence condition. Based on the arousal-mood hypothesis, we hypothesized that the sound environment judged to be the most stimulating and pleasant—the stimulating music condition—would be the most beneficial environment to optimize cognitive performance.

## Materials and Methods

### Participants

46 participants [27 females (58.7% of the sample), mean age: 25.57 years ± 4.33, mean years of education: 17.1 years ± 2.24]. All participants were native French speakers, had normal hearing (measured by a brief hearing test done with an audiometer AC40 Interacoustics; participants had pure tone thresholds under 40 dB SPL; World Health Organization, 1980) and normal or corrected-to-normal vision. None of the participants had color blindness or a history of neurological/psychiatric/neurodevelopmental disorders. None of them were taking drugs or medication that affected the central nervous system during the study. In addition, participants were excluded if they presented at least one of the following criteria: (i) music perception deficits (i.e., performance below 73% at the scale subtest, 70% at the off-beat subtest, and 68% at the out-of-key subtest of the online Montreal Battery of Evaluation of Amusia (MBEA; Peretz and Vuvan, 2017); (ii) presence of mood disorders, evaluated with the Beck Depression Inventory-II (BDI-II; Beck et al., 1996) and the Beck Anxiety Inventory (BAI; Beck et al., 1988) and (iii) musicians. Individuals were considered musicians if they completed equal to or more than 5 years of formal music lessons or were self-taught under that time frame in learning/practicing an instrument, and were practicing a musical instrument equal to or more than 2 h per week (Zhang et al., 2020). The average number of years of musical training/practice of the participants (calculated by taking the number of years of formal music training added to the number of years of autodidactic learning or practicing an instrument) was 1.95 years ± 2. All participants gave their written informed consent in accordance with regulation of the local ethics committee at the University of Montreal.

### Auditory Materials

The 16 auditory stimuli encompassed eight musical excerpts and eight acoustically music-matched noise stimuli. The eight musical stimuli (four highly pleasant and stimulating excerpts and four highly pleasant and relaxing excerpts) were selected from our lab database of 42 short instrumental classical music excerpts, all in major mode. The selection was made based on valence (i.e., 0 = very unpleasant—100 = very pleasant), arousal (i.e., 0 = very relaxing—100 = very stimulating) and familiarity judgments (i.e., 0 = unknown—100 = very familiar) obtained by 46 non-musicians who did not participate in the current study; using visual analog scales (Nadon et al., 2016; for more information see Supplementary Material). The original excerpts from our database consisted of the first 30 s of each piece of music. In order to be able to accumulate enough data for each musical excerpt during the experimental task, the excerpts for the current study were made up of the first 60 s of the same musical pieces. Using data from Nadon et al. (2016), independent-samples *t*-tests revealed that excerpts in the relaxing condition differ significantly from the ones in the stimulating condition in terms of arousal (respectively, *M* = 11.73 ± 11.1, *M* = 79.18 ± 18.75; *t*_(__366__)_ = −42, *p* < 0.001, η^2^ = 0.82) and familiarity (respectively, *M* = 44.35 ± 36.72, *M* = 91.35, ± 19.25; *t*_(__366__)_ = –15.38, *p* < 0.001, η^2^ = 0.39). No difference of valence was found between the relaxing and stimulating excerpts (respectively, *M* = 80.61 ± 18.72, *M* = 78.43 ± 21.11; *t*_(__366__)_ = –1.1, *p* = 0.3, η^2^ = 0.01 (see Supplementary Material for more information).

For auditory control conditions, acoustically music-matched noises were created based on the signal-processing procedure used in previous research (Zatorre et al., 1994; Blood et al., 1999). The spectral envelope of each music stimulus was exported and applied to a synthesized white noise. This generated “noise melody” was thus different for each matched music stimuli. To ensure that participants would not recognize the rhythmic patterns from the matched music piece while listening to the music-matched noise stimulus, each noise stimulus was played in reverse to create the final music-matched noise stimulus.

The final 16 stimuli (i.e., eight musical excerpts and eight acoustically music-matched noise excerpts) were normalized in amplitude, had a duration of 60 s, with 1 s ms fade-in and 2 secfade-out. All above sound processing was performed using Adobe Audition 3.0 software (Adobe Systems Inc., San Jose, CA, United States).

### Experimental Stroop Task

Participants performed the task in a soundproof room. Visual information was displayed on a computer monitor at a distance of 60 cm, while auditory information was presented binaurally using headphones (DT770 Pro, Beyerdynamic) at a sound level ranging approximately around 60 decibels. This decision was made based on the results of Thompson et al. (2011) in which music demonstrated a deleterious effect on reading comprehension performance when presented at around 72 decibels, mainly for fast tempo music, compared to when presented at 60 decibels. Based on these results, 60 decibels appears to be the ideal sound level to perform a cognitive task simultaneously. Participants had access to a keyboard and a mouse, all of which were connected to the computer (HP ProDesk 600 G1, Windows 7) located outside the room, on which the task was run. Communication between inside and outside the soundproof room was done using microphones.

**FIGURE 1:**
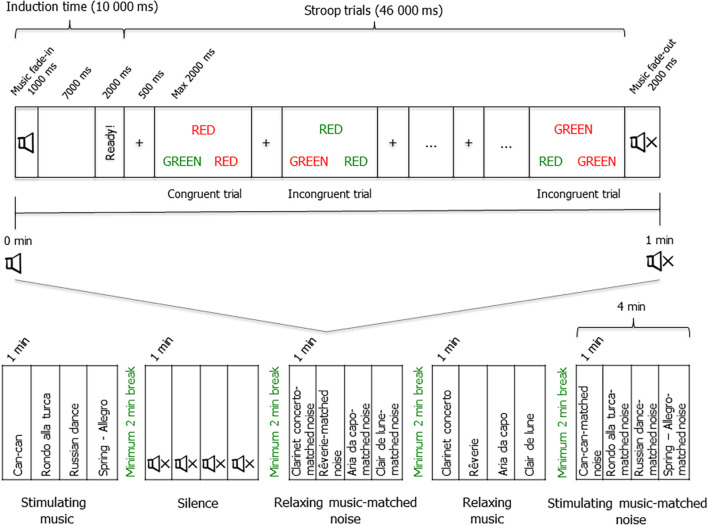
Schematic representation of the experimental procedure and experimental Stroop block.

Selective attention was measured using a computerized Stroop-type task (Stroop, 1935; customized scripted and inspired by the Double trouble task from the *Cambridge Brain Sciences team*^1^). Each trial presented a target word (RED or GREEN) that appeared above two response words (RED and GREEN, see Figure 1). The color of the target word was either congruent (e.g., the word RED presented in red ink) or incongruent (e.g., the word RED presented in green ink) to the meaning of the word. To add a level of difficulty, when the trial was incongruent, the ink color in which the response words were presented was also incongruent. Participants therefore had to identify the ink color of the target word by selecting (with the keyboard arrows left and right) the correct response word presented underneath. The presentation of stimuli, and the recording of the type of stimuli presented, response time and accuracy, were carried out using the Psychtoolbox-3.0.13 (developed by Matlab and GNU Octave) implemented in Matlab 2015b (Mathworks Inc., Natick, MA, United States).

Participants were instructed to perform Stroop trials while being as fast and accurate as possible. Each Stroop trial consisted of a fixation cross (presented 500 ms, see Figure 1) followed by one of the eight possible color-word stimulus options, presented in a pseudo-randomized order. Participants had a maximum of 2,000 ms to give their answer. If participants answered before this given time, another trial began and so on. Past this time, the trial ended, a missed trial was recorded, the words “Too late” appeared on the screen (for 400 ms), and the next trial began.

### Procedure

Participants practiced performing the task in three blocks of 30 s, with a possibility to take a break between the blocks in order to clarify instructions if needed. Each block was performed, respectively, in silence; accompanied by a music stimulus previously judged to have a neutral level of activation and high level of valence (see Supplementary Table 1; Nadon et al., 2016), and with the matched noise stimulus. Practice blocks were similar to experimental blocks, except that the participants responded to Stroop trials for only 16 s. During these practice blocks, participants received feedback for their answers (correct/incorrect; for 800 ms). After completing the three practice blocks, participants could choose to receive the instructions specific to the experimental part, or to continue practicing (by performing all three blocks again).

For the experimental testing, participants performed the Stroop task in five sound conditions: Silence (S), relaxing music (RM), relaxing music-matched noise (RMN), stimulating music (SM), and stimulating music-matched noise (SMN; see Figure 1). The order in which participants performed the sound conditions was counterbalanced across participants and the order of presentation of musical or noise stimuli inside the same sound condition was randomized across participants using the Matlab script. Each sound condition consists of four consecutive blocks of 60,000 ms. Each block began with an induction phase (for 8,000 ms) presenting a blank screen while the participant either listened to the music or noise played, or remained in silence, depending on the sound condition that was performed (see Figure 1). Then, the word “Ready!” was presented (for 2,000 ms), followed by the beginning of a 46,000 ms sequence of Stroop task trials. Participants therefore performed their last Stroop trial just before the sound fade-out, when applicable. When participants completed a sound condition (total of 4 min), they had to take a break of at least 2 min, during which they left the soundproof room to fill out the questionnaires, until they were asked to return to the room to perform the next condition.

After completing the task, participants were asked to listen to each auditory stimulus they heard during the task. Stimuli were presented in a randomized order and visual analog scales were showed on the screen. Participants were asked to evaluate the level of arousal [very relaxing (0) to very stimulating (100)], valence [very unpleasant (0) to very pleasant (100)], and familiarity [unknown (0) to very familiar (100)] for each auditory stimulus.

### Data Analyses

Accuracy (error rate (ER); percentage of incorrect responses excluding missed trials) and mean response times (RT) of correct responses trials were computed for each participant, for each sound condition (i.e., RM, NRM, SM, NSM, and S) and Stroop congruence trial type (i.e., congruent and incongruent). A trial was considered correct when the participant was able to accurately identify the ink color of the target word within the imposed time limit (2,000 ms). Of these correct trials, a first mean and standard deviation were calculated, and only RT between −1.97 and 1.97 standard deviation from the participant’s mean were used to calculate mean RT. The Stroop interference effect was calculated by subtracting mean RT of congruent from incongruent conditions (i.e., mean RT incong.—mean RT cong.) for each sound condition. Mean ER and RT were entered into separate repeated measures analyses of variance (ANOVAs) with Sound Conditions and Stroop Congruence trial type as within-subject factors. Mean Stroop interference scores were entered into another repeated measures ANOVA with Sound Conditions as within-subject factor. When interactions or a principal effect were significant, *t*-test analysis were performed.

Paired-sample *t*-tests were performed to evaluate differences between judgments of arousal, valence and familiarity for the musical stimuli and the music-matched noise stimuli (see Supplementary Table 2). All data were analyzed using IBM SPSS Statistics 26 (IBM Corp., 2019). The alpha levels were set at.05 for all analyses.

## Results

### Auditory Stimuli Evaluation

**FIGURE 2:**
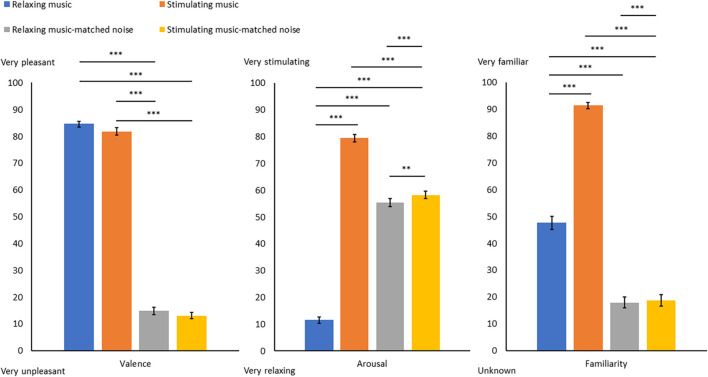
Mean scores for the emotional judgments of valence and arousal and mean scores for the evaluation of familiarity for each sound condition. Graph shows standard errors. ^∗∗^*p* < 0.01. ^∗∗∗^*p* < 0.001.

As expected, judgments of arousal from participants were significantly higher for the stimulating music (SM) compared to the relaxing music (RM). The arousal was judged significantly higher for the two noise conditions (RMN and SMN) compared to the RM. Similarly, the arousal was judged significantly lower for the two noise conditions (RMN and SMN) than for the SM. SMN was considered significantly more stimulating than RMN (see Figure 2 and Supplementary Table 3 for details on arousal’s results).

For the evaluation of valence, as expected, participants considered that the two music conditions (RM and SM) were significantly more pleasant than the two noise conditions (RMN and SMN). There was no significant difference between the two music conditions (RM and SM) and the two noise conditions (RMN and SMN) in terms of valence (see Figure 2 and Supplementary Table 3 for details on valence’s results).

The most familiar condition was SM, followed by the RM condition, with the other two noise conditions being significantly less familiar (RMN and SMN). There was no difference between the level of familiarity among the two noise conditions (RMN and SMN; see Figure 2 and Supplementary Table 3 for more details).

### Stroop Task

**FIGURE 3:**
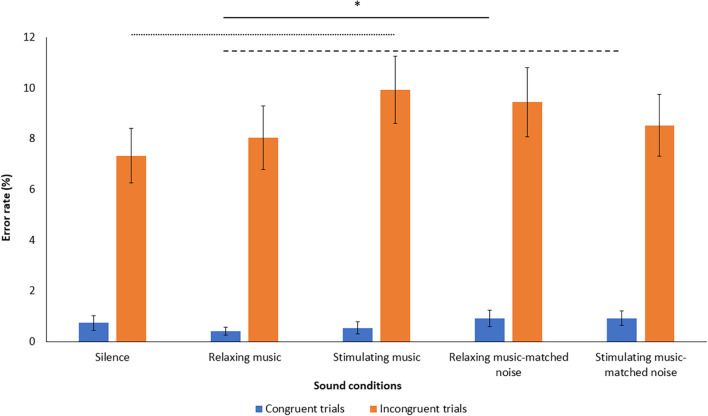
Mean error rate on incongruent and congruent trials for each sound condition. Graph shows standard errors. ^∗^*p* < 0.05. - - - *p* = 0.07. ……*p* = 0.09.

The correct response time (RT) and error rate (ER) analyzes supported the observation of a Stroop interference effect as RTs were significantly slower and ERs were higher on incongruent trials compared to congruent trials [effect of congruence on RTs: *F*_(__1_, _45__)_ = 253.93, *p* < 0.005, η^2^ = 0.85; effect of congruence on error rate: *F*_(__1_, _45__)_ = 104.158, *p* < 0.005, η^2^ = 0.70]. In terms of RT on incongruent and congruent trials, there were no significant differences in performance between the different sound conditions [*F*_(__1_, _45__)_ = 1.01, *p* = 0.405, η^2^ = 0.02]. In the analysis of ERs for incongruent and congruent trials in each sound condition, the ER for congruent trials in the RMN condition was significantly higher than in the RM condition [*t*_(__45__)_ = 2.10, *p* < 0.05, η^2^ = 0.09]. A similar tendency is noted between the ER for congruent trials in the SMN condition compared to the ER in RM condition [*t*_(__45__)_ = 1.81, *p* = *0.077*, η^2^ = 0.07]. Regarding the ER for incongruent trials, there was a trend toward a higher ER in the SM condition compared to the silence condition [*t*_(__45__)_ = –1.69, *p* = 0.097, η^2^ = 0.06, see Figure 3 and Supplementary Table 4 for more details on Stroop’s task results]. No significant effect was found in the analysis with the mean Stroop interference effect scores for each sound condition [*F*_(__1_, _45__)_ = 0.394, *p* = 0.813, η^2^ = 0.009].

## Discussion

The aim of this study was to investigate the effect of the arousal level of background music on selective attention of adults. The results there did not reveal any significant differences in attentional performance depending on whether the task was performed in silence, accompanied by relaxing music or stimulating music. Even though the results showed that participants tended to make a greater number of errors when listening to stimulating music compared to silence, this difference was not significant. However, when comparing on-task performance in the presence of music or noise, performance is more affected by the presence of noise given that there is a significant difference in error rate for congruent trials between relaxing music (RM) and relaxing music-matched noise (RMN), and a trend between relaxing music and relaxing music-matched noise (SMN).

These results are somewhat encouraging as they showed that the addition of to-be processed cognitive information (e.g., background music/noise) does not necessarily have deleterious effects on attentional performance as some theories suggests (e.g., Kahneman (1973) limited capacity model). With these results, it is possible to assume that performing a task requiring attention in the presence of instrumental music should not have a negative effect on the level of selective attention demand in order to perform the task optimally.

The arousal-mood hypothesis (Nantais and Schellenberg, 1999; Thompson et al., 2001, 2011; Husain et al., 2002; Schellenberg and Hallam, 2005; Schellenberg et al., 2008) suggests that a sound environment judged to be stimulating and pleasant would be a beneficial environment to optimize cognitive performance (for details, see Schellenberg, 2013). It was therefore expected that the stimulating music condition would be the sound environment in which we would see the lowest error rate and weakest Stroop interference. In contrast to the hypotheses, the presence of pleasant and stimulating music during the accomplishment of the task did not significantly improve task performance. A small tendency to make more errors on incongruent trials in this sound environment was also noted. These results differ from those of previous work studying the effect of the arousal level of background music upon selective attention (Fernandez et al., 2019; Cloutier et al., 2020). However, these studies mainly aimed to make comparisons between groups (elderly vs. young adults), while the present study had an objective of generalization to the adult population. Furthermore, the tasks involved were different: while previous studies employed the Flanker task to assess selective visual attention, the current study utilized the Stroop task which involves language processing. On the other hand, the number of sound conditions in this study may affect the statistical power of the results. It would therefore be interesting to investigate whether the results would be the same with even a larger sample-size in future studies (even though our sample-size was larger than in previous studies).

A key finding of this study is a negative effect of music-matched noise stimuli (low pleasantness) on attentional performance. These results converge with previous work by Masataka and Perlovsky (2013) and Slevc et al. (2013) showing lower performance on a similar Stroop task in the presence of dissonant music (sound pairings perceived as generally unpleasant or possessing low-pleasantness valence). Interestingly in these studies, greater consonance (sound pairings perceived as generally pleasant or possessing a high-pleasantness valence) led to better performance on the Stroop task. It would therefore be interesting to investigate further to assess which factor, the level of valence/pleasantness or the degree of consonance, had the greater influence upon the results of this and previous studies (Masataka and Perlovsky, 2013; Slevc et al., 2013). This could be done by integrating stimuli that are both consonant and unpleasant, such as scary or sad music, or by specially composed music material. In previous research, the relationship between background music and cognitive performance seems to be affected by the degree of familiarity of the musical stimulus (if the music was already known to the participant). Higher familiarity has a positive effect on performance for cognitive tasks (Darrow et al., 2006; Speer, 2011; Giannouli, 2012). One potential limitation of this study is that, despite an attempt to select equally familiar music of similar valence, the stimulating musical stimuli were rated as more familiar by the participants than the relaxing musical stimuli (see Supplementary Materials for details). It is then surprising that the present findings did not support an effect of stimulating music on task performance given that the stimulating music condition was biased toward higher familiarity.

Judgments of valence can be influenced by the familiarity of a musical piece. Some studies have shown that perceivers tend to find a stimulus that they already know (e.g., a piece of music) more pleasant (Parente, 1976; Schellenberg et al., 2008; Van Den Bosch et al., 2013). Familiar background music has also been associated with increased pleasure in the process of completing a task without compromising task performance (Pereira et al., 2011; Feng and Bidelman, 2015). In this regard, Darrow et al. (2006); Speer (2011), Giannouli (2012), and Kiss and Linnell (2020) asked their participants to bring their favorite music into the lab, which then was used as background music to perform a selective attention task or a sustained-attention task. In these studies, the music selected by the participants held characteristics of high emotional valence and familiarity. However, the other characteristics of the music utilized were heterogeneous between participants (e.g., style, complexity of the music pieces, presence of lyrics, or the level of arousal). The results of these studies indicate that participants consistently performed better in the familiar music conditions. As we know little about the characteristics of the different pieces of music used in these studies and that a great variability is present between them, it is difficult to identify whether the results are generalizable to listening to background music in general or whether they are specifically attributable to a modulation of mood and/or arousal due to the emotional characteristics and familiarity of the music used. Future research should combine this approach with systematic acoustic as well as musical and linguistic structure analyses of the used material to further our understanding of the potential characteristics involved in the observed effects.

Taken together, our findings suggest that it is not sufficient for background music to be arousing, pleasant and familiar in order to enhance attentional performance as suggested by the Arousal-mood theory, and that factors related to individual musical taste may be driving the effects found in previous studies.

Finally, based on the results of this study, we can therefore recommend that tasks requiring selective attention can be performed in an environment of silence as well as with pleasant instrumental music. Findings from this study can be extended to practical use in environments with loud or unpleasant intermittent noises (for example open-plan offices or when space for telework must be shared). According to Szalma and Hancock (2011), intermittent unpredictable short noise bursts are the most disturbing forms of noise; these could be the sound of a horn outside, the laughter of a colleague in a nearby open-plan office, a family member shutting a door nearby, etc. Listening to music in the background may be an efficient tool, equal to working in silence, for masking unpleasant intermittent noises while maintaining a similar level of selective attention on a given task. In this light, future work comparing the presence of music with pleasant noises (such as waves or waterfall noises) would be interesting to investigate given their potential for masking intermittent noises.

## Data Availability Statement

The raw data supporting the conclusions of this article will be made available by the authors, without undue reservation.

## Ethics Statement

The studies involving human participants were reviewed and approved by the Comité d′éthique de la recherche en arts et en sciences (CÉRAS), University of Montreal, Montreal, QC, Canada. The patients/participants provided their written informed consent to participate in this study.

## Author Contributions

ÉN, BT, and NG contributed equally to the project’s conception and the study design. ÉN performed the literature search. ÉN and AS drafted the manuscript and performed the statistical analysis. BT, NG, and ÉN contributed to the establishment of the Stroop task parameters. AS, NG, and BT provided the critical revisions. All authors contributed to the article and approved the submitted version.

## Conflict of Interest

The authors declare that the research was conducted in the absence of any commercial or financial relationships that could be construed as a potential conflict of interest.

## Publisher’s Note

All claims expressed in this article are solely those of the authors and do not necessarily represent those of their affiliated organizations, or those of the publisher, the editors and the reviewers. Any product that may be evaluated in this article, or claim that may be made by its manufacturer, is not guaranteed or endorsed by the publisher.

## Footnotes

1
www.cambridgebrainsciences.com/tests/double-trouble
