# Supplementary material for: The Emotional Effect of Background Music on Selective Attention of Adults
Source: Front Psychol. 2021 Oct 4;12:729037. doi: 10.3389/fpsyg.2021.729037 (PMC8521063; doi:10.3389/fpsyg.2021.729037)
Supplement: Supplementary file 16 [file Table_1.docx]

Supplementary Material

**Supplementary Table S1**

*Description of musical stimuli*

|  |  |  | Results from the emotional judgment of 46 non-musicians (Nadon et al., 2016) | | | Descriptive information of each stimulus | |
| --- | --- | --- | --- | --- | --- | --- | --- |
|  | Piece title | | Arousal^a^ | Valence^b^ | Familiarity^c^ | Tempo  (bpm)^d^ | Interpret |
| Neutral music for the practice part | | |  |  |  |  |  |
|  | Cello Suite No. 1 in G Major (BWV 1007): 1. Prélude/Bach | | 51.45  (24.1) | 83.88  (22.78) | 87.72  (17.58) | 135 | István Várdai |
| Relaxing music | | |  |  |  |  |  |
|  | Clarinet concerto in A major (K622) – 2. Adagio/Mozart | | 13.28  (12.2) | 78.86  (20.62) | 51.52  (31.68) | 80 | Anthony Pike, English Chamber Orchestra & Ralf Gothóni |
|  | Rêverie/Debussy | | 13.77  (11.7) | 78.34  (18.81) | 35.14  (34.26) | 65 | Rebecca Arons |
|  | Goldberg variations (BWV988) – Aria Da Capo/Bach | | 10.35  (10.35) | 81.26  (17.88) | 33.21  (34.07) | 75 | Nicola Frisardi |
|  | Suite bergamasque – 3. Clair de lune/Debussy | | 9.5  (9.65) | 84  (17.51) | 57.54  (41.24) | 70 | François-Joël Thiollier |
| Stimulating music | | |  |  |  |  |  |
|  | Can-can from Orpheus in the Underworld/Offenbach | | 82.27  (14.52) | 76.21  (22.71) | 89.37  (20.7) | 160 | Charles Gerhardt & London philharmonic orchestra |
|  | Piano Sonata No.11 in A Major (K331) – Rondo: alla turca/Mozart | | 73.33  (23.86) | 84.72  (15.53) | 94.92  (14.71) | 110 | Finghin Collins |
|  | Russian dance from The Nutcracker/Tchaikovsky | | 88.66  (12.42) | 78.01  (19.99) | 86.38  (24.27) | 140 | Heinz Rögner & Berlin Radio Symphony Orchestra |
|  | Concerto No. 1 in E major, Op. 8 (RV 269) – Spring 1. Allegro/Vivaldi | | 72.45  (17.66) | 74.78  (24.44) | 94.74  (14.81) | 115 | Jonathan Carney & Royal Philharmonic Orchestra |

*Note.* This table presents means (and standard deviations) for emotional judgment of arousal, valence and familiarity compiled with a computerized version of visual analog scales. The table shows the data from the previous research by Nadon et al. (2016).

^a^ 0 = very relaxant, 100 = very stimulating

^b^ 0 = very unpleasant, 100 = very pleasant

^c^ 0 = unknown, 100 = very familiar

^d^ bpm = beat per minute

**Supplementary Table S2**

*Participant judgment of auditory stimuli*

|  |  | | Results from the emotional judgment of 46 non-musicians (Nadon et al., 2016) | | | Results from the emotional judgment of 46 non-musicians from this study | | |
| --- | --- | --- | --- | --- | --- | --- | --- | --- |
|  | Stimuli title | | Arousal^a^ | Valence^b^ | Familiarity^c^ | Arousal^a^ | Valence^b^ | Familiarity^c^ |
| Relaxing music and their music-matched noise condition | | | | | | |  |  |
|  | Clarinet concerto in A major (K622) – 2. Adagio | | 13.28  (12.2) | 78.86  (20.62) | 51.52  (31.68) | 17.44  (18.12) | 85.32  (12.68) | 60.28  (31.32) |
|  | Clarinet concerto-matched noise | |  |  |  | 56.47  (21.21) | 15.08  (19.33) | 17.88  (29.03) |
|  | | Mean comparison between music and music-matched noise |  |  |  | -9.59*** | 20.64*** | 8.79*** |
|  | Rêverie | | 13.77  (11.7) | 78.34  (18.81) | 35.14  (34.26) | 12.91  (18.05) | 82.96  (14.95) | 33.28  (25.12) |
|  | Rêverie-matched noise | |  |  |  | 57.84  (21.09) | 11.03  (15.34) | 19.90  (28.26) |
|  | | Mean comparison between music and music-matched noise |  |  |  | 22.95*** | -10.67*** | 2.6* |
|  | Goldberg variations (BWV988) – Aria Da Capo | | 10.35  (10.35) | 81.26  (17.88) | 33.21  (34.07) | 9.83  (13.17) | 81.89  (15.79) | 30.62  (27.42) |
|  | Aria da capo-matched noise | |  |  |  | 54.42  (20.36) | 19.29  (22.06) | 16.89  (27.16) |
|  | | Mean comparison between music and music-matched noise |  |  |  | -12.57*** | 17.84*** | 2.56* |
|  | Suite bergamasque – 3. Clair de lune | | 9.5  (9.65) | 84  (17.51) | 57.54  (41.24) | 6.16  (9.16) | 88.00  (15.24) | 66.49  (33.36) |
|  | Clair de lune-matched noise | |  |  |  | 52.89  (21.61) | 14.05  (18.27) | 17.04  (28.54) |
|  | | Mean comparison between music and music-matched noise |  |  |  | -13.38*** | 20.32*** | 8.98*** |
| Stimulating music and their music-matched noise condition | | | | | |  |  |  |
|  | Can-can from Orpheus in the Underworld | | 82.27  (14.52) | 76.21  (22.71) | 89.37  (20.7) | 81.67  (15.37) | 77.54  (19.49) | 84.01  (22.13) |
|  | Can-Can-matched noise | |  |  |  | 56.28  (19.36) | 15.03  (16.38) | 17.95  (28.85) |
|  | | Mean comparison between music and music-matched noise |  |  |  | 7.9*** | 16.87*** | 14.45*** |
|  | Piano Sonata No.11 in A Major (K331) – Rondo: alla turca | | 73.33  (23.86) | 84.72  (15.53) | 94.92  (14.71) | 75.73  (22.01) | 83.78  (20.49) | 95.98  (7.67) |
|  | Rondo: alla turca-matched noise | |  |  |  | 60.70  (18.02) | 11.95  (15.16) | 17.64  (27.92) |
|  | | Mean comparison between music and music-matched-noise |  |  |  | 3.02** | 18.0*** | 19.19*** |
|  | Russian dance from The Nutcracker | | 88.66  (12.42) | 78.01  (19.99) | 86.38  (24.27) | 84.99  (16.24) | 83.47  (19.74) | 89.36  (18.89) |
|  | Russian dance-matched noise | |  |  |  | 57.97  (18.19) | 13.64  (16.59) | 22.40  (31.62) |
|  | | Mean comparison between music and music-matched noise |  |  |  | 8.22*** | 17.49*** | 14.65*** |
|  | Concerto No. 1 in E major, Op. 8 (RV 269) – Spring 1. Allegro | | 72.45  (17.66) | 74.78  (24.44) | 94.74  (14.81) | 75.28  (20.02) | 82.37  (18.59) | 96.05  (6.42) |
|  | Spring – Allegro-matched noise | |  |  |  | 57.92  (20.12) | 11.64  (14.91) | 16.43  (27.13) |
|  | | Mean comparison between music and music-matched noise |  |  |  | 4.66*** | 18.23*** | 19.7*** |

*Note.* For the music and music-matched noise rows, this table shows means (and standard deviations) for emotional judgments of arousal, valence and familiarity compiled with a computerized version of visual analog scales. The table presents the data from previous research by Nadon et al. (2016) and the new data from the current study using the same musical excerpts. For the mean comparison between music and music-matched noise rows, paired-samples t-tests were performed to compare mean values for emotional judgments of valence, arousal and familiarity for all conditions and scores presented are t-scores.

^a^ 0 = very relaxant, 100 = very stimulating

^b^ 0 = very unpleasant, 100 = very pleasant

^c^ 0 = unknown, 100 = very familiar

**p* < .05. ***p* < .01. ****p* <.001.

**Supplementary Table S3**

*Comparisons between judgments of valence, arousal, and familiarity for each sound condition*

|  | | df | *t* | *p* | Effect size (η^2^) |
| --- | --- | --- | --- | --- | --- |
| Valence | |  |  |  |  |
| Relaxing music/Stimulating music | 183 | 1.57 | =0.119 | =0.01 |  |
| Relaxing music/Relaxing music-matched noise | 183 | 40.25 | =0.000 | =0.90 |  |
| Relaxing music/Stimulating music-matched noise | 183 | 44.33 | =0.000 | =0.92 |  |
| Stimulating music/Relaxing music-matched noise | 183 | 31.31 | =0.000 | =0.84 |  |
| Stimulating music/Stimulating music-matched noise | 183 | 35.13 | =0.000 | =0.87 |  |
| Relaxing music-matched noise/Stimulating music-matched noise | 183 | 1.74 | =0.084 | =0.02 |  |
| Arousal | |  |  |  |  |
| Relaxing music/Stimulating music | 183 | -36.89 | =0.000 | =0.88 |  |
| Relaxing music/Relaxing music-matched noise | 183 | -22.46 | =0.000 | =0.73 |  |
| Relaxing music/Stimulating music-matched noise | 183 | -24.78 | =0.000 | =0.77 |  |
| Stimulating music/Relaxing music-matched noise | 183 | 11.46 | =0.000 | =0.42 |  |
| Stimulating music/Stimulating music-matched noise | 183 | 11.12 | =0.000 | =0.40 |  |
| Relaxing music-matched noise/Stimulating music-matched noise | 183 | -3.01 | =0.003 | =0.05 |  |
| Familiarity | |  |  |  |  |
| Relaxing music/Stimulating music | 183 | -16.84 | =0.000 | =0.61 |  |
| Relaxing music/Relaxing music-matched noise | 183 | 10.30 | =0.000 | =0.37 |  |
| Relaxing music/Stimulating music-matched noise | 183 | 9.77 | =0.000 | =0.34 |  |
| Stimulating music/Relaxing music-matched noise | 183 | 34.01 | =0.000 | =0.86 |  |
| Stimulating music/Stimulating music-matched noise | 183 | 33.11 | =0.000 | =0.86 |  |
| Relaxing music-matched noise/Stimulating music-matched noise | 183 | -0.75 | =0.454 | =0.00 |  |

*Notes.* This table presents the results for paired t-test analysis.

**Supplementary Table S4**

*Means and standard deviations for all study variables by sound condition*

|  | Sound conditions | | | | |
| --- | --- | --- | --- | --- | --- |
| Variables | Silence | Relaxing music | Stimulating music | Relaxing music-matched noise | Stimulating music-matched noise |
| Congruent Stroop RT (ms) | 842.67 (168.67) | 839.05 (156.60) | 856.64 (120.23) | 843.63 (147.86) | 836.15 (154.28) |
| Incongruent Stroop RT (ms) | 1093.47 (192.56) | 1075.64 (170.17) | 1096.68 (168.50) | 1083.66 (180.23) | 1081.05 (181.72) |
| Stroop Interference Effect (ms) | 251.02 (121.50) | 236.43 (123.84) | 240.11 (116.00) | 239.96 (95.66) | 240.11 (123.95) |
| ER: Congruent Stroop (%) ^a^ | 0.74 (1.87) | 0.41 (1.10) | 0.54 (1.66) | 0.92 (2.21) | 0.93 (1.93) |
| ER: Incongruent Stroop (%) ^b^ | 7.33 (7.33) | 8.04 (8.47) | 9.92 (8.98) | 9.44 (9.20) | 8.52 (8.28) |
| Unsuccessful rate (%) ^c^ | 5.72 (5.62) | 6.18 (6.39) | 7.00 (6.11) | 6.70 (5.84) | 6.01 (5.55) |

*Note.* This table shows mean (and standard deviations) values. Data in the first three rows are in milliseconds (ms) and data in the last three rows are in percentages; see information below for more details.

^a^ Error rate: Congruent Stroop: failed trials for congruent trials/total number of congruent trials (failed + successful)

^b^ Error rate: Incongruent Stroop: failed trials for incongruent trials/total number of incongruent trials (failed + successful)

^c^ Unsuccessful rate: missed trials + failed trials (wrong answer)/total number of trials (missed + successful + failed)
